# Long non-coding RNA expression in PBMCs of patients with active pulmonary tuberculosis

**DOI:** 10.3389/fmicb.2023.1257267

**Published:** 2023-12-14

**Authors:** Guoli Li, Zhelong Feng, Honghuan Song, Yajing Wang, Limei Zhu, Yan Li

**Affiliations:** ^1^Integrated Service and Management Office, Jiangsu Provincial Center for Disease Control and Prevention, Nanjing, China; ^2^Department of Chronic Communicable Disease, Jiangsu Provincial Center for Disease Control and Prevention, Nanjing, China; ^3^School of Basic Medicine and Clinical Pharmacy, China Pharmaceutical University, Nanjing, China

**Keywords:** tuberculosis, latent tuberculosis infection (LTBI), long non-coding RNAs, PBMCs, RRT-PCR, biomarker

## Abstract

**Purpose:**

*Mycobacterium tuberculosis (Mtb)* infection is the primary cause of the chronic infectious illness tuberculosis (TB). Long non-coding RNAs (lncRNAs) are functional RNA molecules that cannot be translated into proteins and play a crucial role in regulating the immune system’s innate and adaptive responses. It has been demonstrated that the dysregulation of lncRNA expression is associated with various human diseases. However, the mechanism underlying the involvement of so many lncRNAs in the immune response to TB infection remains unclear. The objective of our current study was to identify a number of significantly differentially expressed lncRNAs in peripheral blood mononuclear cells (PBMCs) from TB patients and to select the most indicative lncRNAs as potential biomarkers for active pulmonary tuberculosis.

**Methods:**

Microarray analysis was performed to determine the lncRNA and mRNA expression profiles in TB patients using a case-control model. The differentially expressed lncRNAs were subjected to gene ontology (GO) and Kyoto Encyclopedia of Genes and Genomes (KEGG) pathway analysis to investigate potential roles and pathways associated with the pathogenesis of TB infection, and to screen lncRNAs specifically linked to TB infection. Using real-time fluorescence quantitative PCR (QRT-PCR), specific lncRNAs were identified in TB patients and latent infections.

**Results:**

Our findings revealed that various signaling pathways were differentially expressed in TB-infected individuals, suggesting a potential role for lncRNAs in the immunological responses driven by TB infection. This study provides crucial guidelines for future functional research. Upregulated lncRNAs were mainly enriched in Neutrophil extracellular trap formation and Chemokine signaling pathways, while downregulated lncRNAs were enriched in Neuroactive ligand-receptor interaction and Cushing syndrome in TB PBMCs. Furthermore, we found that lnc-XPNPEP1-5, lnc-CASKIN2-2, lnc-HSPA13-6, lnc-CLIC5-1, and LINC02502 were significantly downregulated in TB-infected patients, while LINC00528, lnc-SLC45A4-3, and LINC00926 were significantly upregulated in TB patients and latent infections. These eight lncRNAs, identified as novel biological marker candidates for diagnosing TB infection, were validated by real-time fluorescence quantitative PCR (QRT-PCR).

**Conclusion:**

The abnormally expressed lncRNAs identified in this research may provide crucial information for understanding the pathophysiological characteristics of TB patients and the dysfunction of PBMCs. Our findings reveal potential targets for early TB diagnosis and therapy, as well as offer new insights into the mechanisms underlying TB infection.

## Introduction

Tuberculosis (TB) is a grave public health concern that poses significant risks to human health. *Mycobacterium tuberculosis (Mtb)*, the pathogen responsible for TB, is prevalent among approximately one-third of the global population. Among those infected, roughly 5–10% suffer from active pulmonary tuberculosis. The World Health Organization’s (WHO) most recent study estimates that there were 10.6 million new TB cases worldwide in 2021, with an incidence rate of 134 per 100,000. Globally, TB remains one of the leading causes of mortality, and in 2021 it was the second-highest cause of death overall, surpassed only by COVID-19. China ranks third among the 30 nations with the highest TB burden, following India and Indonesia. In 2021, China reported 780,000 new TB cases, with a tuberculosis incidence of 55 per 100,000 individuals. The increasing incidence of multidrug-resistant (MDR), extensively drug-resistant (XDR), and entirely drug-resistant TB is exacerbating the challenges associated with this disease (World Health Organization, 2022). To prevent and control TB and develop novel medications, it is essential to understand the mechanisms of *Mtb* infection and survival within the host (Boro et al., 2016). Following macrophage phagocytosis during host-pathogen interactions, *Mtb’s* intracellular survival and reproduction elicit an immunological response that ultimately leads to the development of granulomas and/or disease manifestations (Qian et al., 2018). To facilitate the eradication of active tuberculosis (ATB), host cells have evolved several clearance processes, which include apoptosis, autophagy, inflammation, and macrophage polarization. Concurrently, *Mtb* has developed an array of near-perfect immune evasion mechanisms to help it evade the host immune system (Boro et al., 2016; Healy et al., 2021).

Long-non-coding RNAs (lncRNAs) are a type of RNAs with transcripts longer than 200 nucleotides that do not directly encode proteins but have the ability to regulate chromatin remodeling and transcriptional and post-transcriptional regulation of gene expression (Schwenk and Arnvig, 2018; Li Y. et al., 2019). At the epigenetic, transcriptional, and post-transcriptional levels, lncRNAs have been demonstrated to play crucial roles in controlling gene expression. LncRNAs exert their influence on a broad range of biological processes through intricate and diverse mechanisms, such as participating in the regulation of chromatin’s open or closed state prior to transcription, interacting with transcription factors to modulate gene transcription, and engaging in variable shear regulation (Schwenk and Arnvig, 2018; Wang et al., 2019; Fathizadeh et al., 2020). LncRNAs possess the capacity to form hybrid structures with DNA, thereby affecting gene expression. They interact with unstranded DNA bases through complementary pairing to control DNA methylation and transcriptional inhibition (Zhang et al., 2019). Additionally, lncRNAs can alter the binding of transcription factors at promoter regions, impeding their activity or limiting the recruitment of Pol II, thereby modifying the transcription of downstream target genes (Wang and Chang, 2011; Yang et al., 2016; Li Y. et al., 2019). LncRNAs attract transcription regulators to the promoter region of target genes at the RNA level, where they exert control over the target genes’ transcription (Ye et al., 2022; Zhang et al., 2022). At the protein level, lncRNAs bind to specific proteins to govern their associated protein activity or modify the cellular localization of these proteins.

According to accumulating evidence, lncRNAs significantly regulate the innate immunological response of host macrophages (Li M. et al., 2019). LncRNAs participate in innate immunity that is regulated by macrophages, and an inducible pattern of inflammatory gene expression is essential for the body’s defense against microbes (Hu et al., 2020; Li et al., 2020; Wang et al., 2020). For instance, to control the inflammatory response to *Mtb* infection, lincRNA-Cox2 regulates the activation or repression of immune-related genes, activating NF-κB and STAT (Schwenk and Arnvig, 2018; Yan et al., 2018). Numerous studies indicate that a significant number of lncRNAs likely play a vital role in the pathophysiology of tuberculosis by controlling the apoptosis and autophagy caused by *Mtb* in macrophages (Roy et al., 2018). For example, macrophages infected with BCG induce apoptosis by upregulating the expression of lincRNA-Cox2. Activation of the PERK-eIF-2-CHOP signaling pathway by Cox2 knockdown exacerbates the accumulation of reactive oxygen species (ROS) and initiates apoptosis (Yan et al., 2018).

Most clinical diagnostic techniques currently in use have certain limitations (Healy et al., 2021; Wei et al., 2021). Interferon-gamma release assays (IGRA) are unable to differentiate between latent tuberculosis infection (LTBI) and active tuberculosis (ATB), while sputum smear and culture lack adequate sensitivity, timeliness, and a poor detection rate for smear-negative pulmonary tuberculosis (PTB) (Venkatappa et al., 2019). The World Health Organization (WHO) has recently proposed non-pathogen-based diagnosis as a method to enhance the rapid and universal identification of clinically confirmed TB (Broderick et al., 2021; Jung et al., 2021). As such, biomarkers of the host immune response may provide critical information for addressing this issue (Li et al., 2020; Fang et al., 2021). Various lncRNAs have been proposed as potential biomarkers. An increasing body of research indicates that peripheral blood mononuclear cells (PBMCs) from TB patients exhibit abnormal expression of lncRNAs (Qian et al., 2018; Zhang et al., 2020; Yi et al., 2021). A study suggests that two highly aberrantly expressed long non-coding RNAs (MIR3945HG V1 and MIR3945HG V2) in PBMC samples from individuals with active PTB could potentially serve as novel diagnostic biomarkers (Kaushik et al., 2021). Additionally, NEAT1 expression—including both NEAT1 1 and NEAT1 2—was found to be elevated in TB patients compared to healthy controls, with the expression declining progressively throughout therapy and returning to normal levels. This dynamic change in NEAT1 expression could potentially indicate the effectiveness of anti-TB treatment, making it a possible biomarker for prognosticating TB patient outcomes (Huang et al., 2018). In this study, we employed microarray detection to identify multiple key lncRNAs that are expressed in PBMCs of TB patients. By selecting the most representative lncRNAs, we aim to establish them as potential biomarkers for active pulmonary tuberculosis. Further research is required to explore the mechanism of these lncRNAs in the immune imbalance that occurs in pulmonary tuberculosis. This understanding may provide valuable insights into their role in the pathogenesis of the disease and could help guide therapeutic interventions. These lncRNAs could serve as biomarkers for biological early warning and precision treatment, enabling more accurate diagnosis, prognosis, and personalized treatment plans for TB patients. This approach has the potential to improve patient outcomes and reduce the burden of tuberculosis worldwide.

## Materials and methods

### Study cohort

The study group comprised of three subgroups: TB patient group, TB infection group, and healthy control group (HC group).

Tuberculosis patient group: 53 TB patients receiving primary therapy who tested pathogenic positive were enrolled from the Department of TB at Jiangsu Infectious Disease Hospital. All patients were diagnosed according to clinical manifestations, bacteriological identification, and genotyping as *Mycobacterium tuberculosis*, in accordance with the 2017 Chinese Medical Association’s Clinical Diagnosis and Treatment Manual (Tuberculosis Branch). The study was approved by the Jiangsu Provincial Center for Disease Control and Prevention, and all patients provided informed consent (Ethics Authorization Number: JSCDCLL (2014)003).

Tuberculosis infection group: Blood specimens from the same period of physical examination, identified as TB infection by the IGRA (T-spot) method, constituted a total of 50 cases. The T-spot Positive result was defined as having ≥8 spots in Panel A and/or Panel B.

HC group: Blood specimens from the same period of physical examination, identified as non-TB infected by IGRA (T-spot) method, included a total of 53 cases. The T-spot Negative result was defined as having both Panel A and Panel B spot counts ≤4, including values <0.

The differences in age and sex between the above 3 groups were not statistically significant (*P*-value > 0.05). For the microarray analysis, three samples from the HC group and three samples from the TB Patient group were included. The remaining samples in each group were used for validation (50 samples in each group).

### Reagents and instruments

Erythrocyte lysate was purchased from Beijing Tiangen Biotechnology Co., Ltd. Ficoll lymphocyte separation solution was purchased from Sigma Company, and Trizol, Superscript III PlatinumOneStep fluorescent quantitative PCR System reagents were purchased from Invitrogen Company. DX fluorescent quantitative PCR instrument was purchased from ABI company, AII biosafety cabinet was purchased from Thermo company, and CO_2_ incubator was purchased from Thermo Fisher.

### PBMC collection

In this study, 5 mL of venous blood was collected from research subjects using Vacutainer^®^ CPT™ Mononuclear Cell Preparation Tube (BD, Cat. #362761), which could separate mononuclear cells from whole blood in a fully closed system. Whole blood is drawn directly into the CPT™ tubes using standard phlebotomy techniques and processed in the same tube. To isolate the mononuclear cells, the blood samples were centrifuged within 2 h at room temperature at a speed of 1800 RCF for 20 min. During centrifugation, the gel forms a physical barrier between the mononuclear cells in plasma and the erythrocytes and granulocytes. After centrifugation, the collected mononuclear cells were transferred to 15 mL tubes using a Pasteur pipette. To wash the cells, PBS was added and the mixture was centrifuged for 15 min at 300 RCF. Finally, the cell pellet was resuspended in 1 mL of AIM-V medium for further procedures.

### Total RNA extraction

For each RNA preparation, 500 μl of resuspended PBMCs (peripheral blood mononuclear cells) were used. In accordance with the instructions, total RNA was isolated using the mirVana™ RNA Isolation Kit (QIAGEN). The process began by adding an equal volume of chloroform and centrifuging at 10,000 × *g* for 5 min. The supernatant was then collected. Next, 1.25 times the volume of 100% ethanol was added, the mixture was vortexed, and it was passed through a purification column. The centrifuge was run at 10,000 × *g* for 15 s, and 350 μL of wash solution was added. The centrifuge was run again at 10,000 g for 15 s. After that, 10 μL of DNase I and 70 μL of Buffer RDD were added, and the mixture was allowed to stand for 15 min at room temperature. It was then washed 2–3 times with 500 μL, with each wash followed by a centrifuge run at 5–10 s. The purification column was washed twice, and the centrifuge was run at 10,000 g for 15 s. The filtrate was discarded, and the centrifuge was run for 1 min. Lastly, 100 μL of pre-warmed eluent at 95°C was added to the center of the column. The centrifuge was run at the highest speed for 30 s at room temperature, and the collected total RNA was stored in a new EP tube at −80°C.

### Total RNA and chip quantification

Thermo Scientific’s NanoDrop ND-2000 was utilized for RNA quantification, while the Agilent Bioanalyzer 2100 was employed to assess the integrity of the RNA samples (both from Agilent Technologies). The process followed the manufacturers’ recommended protocols for sample labeling, microarray hybridization, and washing. Briefly, the whole RNA was first converted to double-stranded cDNA, which was then transcribed into cRNA and tagged with cyanine-3-CTP. The resulting microarray was hybridized with the respective cRNAs. Post-washing, the Agilent Scanner G2505C was used for scanning the arrays.

### mRNA chip detection

The GeneChip Human TranscriptomeArray 2.0 chip was employed to detect changes in lncRNA expression. Six qualified RNAs, judged by their RIN score ≥7 and 28S/18S ratio ≥0.7, were amplified and transcribed into fluorescently labeled cRNA samples. The Agilent Gene Expression Hybridization Kit (Agilent p/n 5188-5242) was used for Fragmentation and Hybridization, with 33045 LncRNA and 30215 mRNA probes (4 replicates per probe per array). Following hybridization, the microarrays were cleaned, fixed, and scanned using a DNA microarray scanner. The Agilent Scanner was used to scan the chip after hybridization, extracting the original signal values. Data normalization and probe filtering were performed prior to screening for differentially expressed genes. At least one group of samples in each comparison group must have 100% of the probes marked as “P” for subsequent analysis. For analysis with biological replicates, the difference in standardized signal values (Fold change) and significant *P*-value from the *T*-test were used for screening, with criteria of Fold change value ≥2.0 and *P*-value ≤ 0.05.

To search for differentially expressed signaling pathways and functionally forecast the function of the differentially expressed lncRNAs, GO analysis and KEGG pathway analysis were performed. These analyses compared target lncRNAs with genes in databases to obtain information about the biological processes, cellular components, and molecular functions and pathways they participate in. R/Bioconductor packages (GOseq2, KEGG.db3) were used for these analyses. The specific analysis process included Gene annotation, Enrichment analysis, and Functional/Pathway clustering.

### Fluorescence quantitative PCR validation

Based on the chip detection results, specific Taqman primers were designed to select 50 healthy controls without *Mtb* infection, 50 latent TB infection, and 50 patients with confirmed pulmonary tuberculosis for correlation analysis. Eight lncRNAs were chosen from the lncRNA profiles due to their strong association with mRNAs that exhibited minimal variation within the group but showed significant differences between groups. By referring to the existing gene nucleotide sequences in the PubMed database, DNASTAR software was employed for homology analysis, and conserved regions were selected. Subsequently, PrimerExpress 3.0 software was used to design primers and probes, respectively. The synthesis of primers and probes was entrusted to Sangon Biotech (Shanghai) Co., Ltd. A fluorescent emitting group FAM was attached to the 5’ end of the probe, while the fluorescent emitting group BHQ1 was attached to the 3’ end of the probe. Table 1 presents the primer and probe sequences. The target fragment was amplified using a one-step method. The concentrations of primers and probes are presented in Table 1, and a 10 μL reaction system was employed. The system preparation is detailed in Table 2. The conditions for fluorescence quantitative PCR reaction were as follows: 52°C for 10 min, 95°C for 5 min; 95°C for 15 s, 58°C for 40 s, 42 cycles; and 25°C for 5 min. The fluorescence signal was detected at the annealing stage at 58°C. Six replicate wells were prepared for all samples, with GAPDH serving as the internal reference gene. The qRT-PCR results were analyzed based on the Ct value of the sample to be tested, using the relative quantitative method. The relative expression of the target gene (−ΔΔCt) represented the relative change in expression of the target gene in each group of PBMC samples.

**TABLE 1:** Gene hybridization primer and probe sequences and working concentrations.

Gene	Primers/probes (5′-3′)	Final conc. (μM)
FR255266-FP	AACCTACAGAGGCAAATCC	40
FR255266-RP	CCATTAGCTTAGCATCTGTCG	40
FR255266-PROBE	FAM-CTGTAGCCTCATCTCATGTGCT-BHQ1	10
NONHSAT006952-FP	CGCAGCCCTGAAGAAACTAC	40
NONHSAT006952-RP	AAGCTTCACTGTCCCTCCAA	40
NONHSAT006952-PROBE	FAM-GCCGGCTCCTTTGAAAACAGGTCATAT-BHQ1	10
TCONS_l2-FP	AGGTGCCAGTAAATGTATGAG	40
TCONS_l2-RP	TCAGGACTGGTTATGACCG	40
TCONS_l2-PROBE	FAM-AGTGGAGTAGTGCCTCCCTGGT-BHQ1	10
β-Catenin-FP	GCTGCTTGTACGAGCACATCA	40
β-Catenin-RP	TGCGT TCCACCCATGGA	40
β-Catenin-PROBE	FAM-ACACCCAACGGCG-BHQ1	10
TCF-FP	CGCCTGAGGGTCCGAGATA	40
TCF-RP	GCACCATACGGCCAAGCT	40
TCF-PROBE	FAM-CGCGACCCGAATTGAGAACCAAGTAATGCGTCGCG-BHQ1	10
GSK-3β-FP	CCCTCTGGCCACCATCCT	40
GSK-3β-RP	CCCTCTGGCCACCATCCT	40
GSK-3β-PROBE	FAM-CCCTCCACATGCTC-BHQ1	10
GAPDH-FP	ATGGAAATCCCA TCACCATCTT	40
GAPDH-RP	CGCCCCACTTGATTTTGG	40
GAPDH-PROBE	FAM-CAGGAGCGAGATCC-BHQ1	10

**TABLE 2:** The top 10 upregulated and downregulated lncRNAs in the PBMCs of ATB patients, compared to the HC group.

lncRNA gene symbol	Regulation	Fold change	*P*-value
NONHSAG003987	Up	171.35942	0.020535182
NONHSAG028745	Up	119.57172	0.00110843
AD000685.1-001	Up	54.02492	0.000188
RP11-678G14.4-002	Up	51.42291	0.0000638
NONHSAG003812	Up	49.56341	0.001218415
CCDC147-AS1	Up	42.48658	0.007961398
NONHSAG054582	Up	40.38269	0.00755706
FR346361	Up	39.48970	0.00795282
RP4-620F22.2-001	Up	38.01553	0.003028001
RP11-925D8.6	Up	35.73091	0.039328746
linc-SNAP25-1	Down	149.43263	0.005198977
NONHSAG029335	Down	135.94028	0.01675956
NONHSAG015932	Down	112.96288	0.006525897
NONHSAG019454	Down	67.02208	0.000506
LOC101927998	Down	51.443554	0.004187135
linc-GRAMD3-2	Down	33.895023	0.019641997
NONHSAG019533	Down	27.05918	0.010219458
NONHSAG020174	Down	18.319973	0.004563094
NONHSAG041748	Down	17.41906	0.0000936
linc-MRPL22	Down	15.715142	0.00137531

### Statistical analysis

The study results were statistically processed using R Studio version 1.4.1106. The results were expressed as mean ± standard deviation (−x ± s). The *t*-test was employed to compare the means of two groups, while one-way analysis of variance (ANOVA) was used to compare the data between various groups. A *P*-value of less than 0.05 was considered statistically significant.

## Result

### Chip quantification results

**FIGURE 1:**
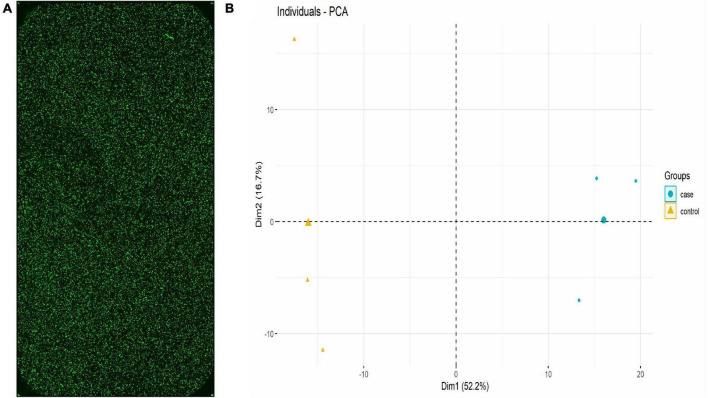
lncRNA expression in each group (Three samples in HC group and three samples in TB patients). **(A)** Chip hybridization scan showed that the fluorescence intensity was good, the signal was clear, and the density was uniform, which confirmed that the hybridization was in good condition. **(B)** PCA plot showed the distribution of the samples. The case and control were concentrated in different regions in the two-dimensional space, indicating that the samples are grouped reasonably and the repeatability within the group was good.

The lncRNA expression in each group was visually represented by the chip hybridization scan, box plot, scatter plot, and Principal Component Analysis (PCA) plot (Figure 1). The chip hybridization scan results demonstrated that the chip hybridization was of good quality, with reliable data signals and distinct expression differences between the two groups. The distribution of data within the case and control groups was more concentrated, and the intergroup difference was more pronounced, indicating that there are significant disparities between the two groups of samples.

### Expression signatures of the deregulated lncRNAs

**FIGURE 2:**
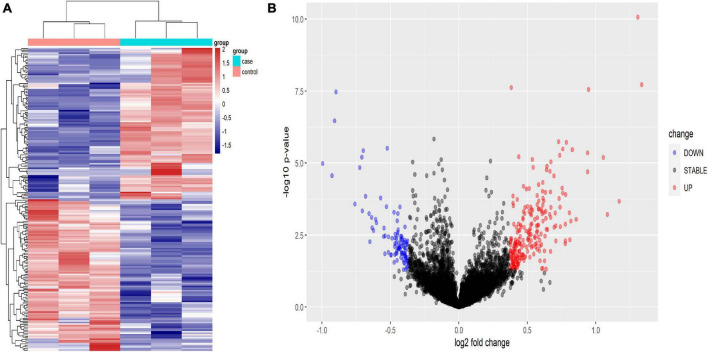
Heat map **(A)** and volcano plot presentation **(B)** of the lncRNA expression profiles in the TB patient group and the HC group. **(A)** Each column represents a sample and each row represents a gene. Red represents high relative expression and blue represents low relative expression. **(B)** Red represents significantly up-regulated genes and blue represents significantly down-regulated genes. Genes with an expression fold change >2 and FDR-adjusted *P*-value < 0.05 were considered statistically significant.

Using human lncRNA arrays, the expression profiles of lncRNAs and mRNAs in peripheral blood mononuclear cells (PBMCs) were investigated. For six samples, the unnormalized raw lncRNA and mRNA data were transformed to log2 values. Unsupervised phylogenetic clustering analysis showed a distinct separation of lncRNAs into two groups (Figure 2A). By applying a fold change of 2 and a false discovery rate (FDR) of 0.05, significantly differentially expressed genes were identified. The volcano plot illustrates the lncRNA abundance and distribution (Figure 2B).

### Expression profile of lncRNAs in PBMCs

**FIGURE 3:**
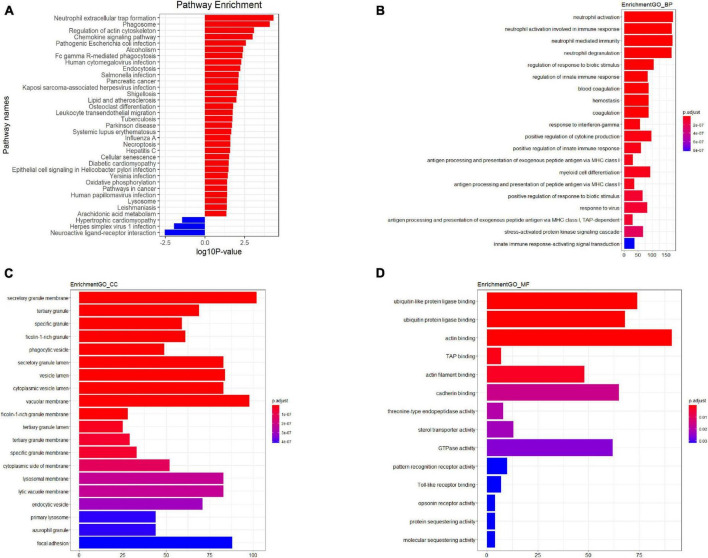
Kyoto Encyclopedia of Genes and Genomes (KEGG) pathway analysis **(A)** GO analysis plot–Biological process **(B)** GO analysis plot–Cellular component **(C)** GO analysis plot–Molecular function **(D)**. *P*-value denoted the significance of the GO term and pathway correlated to the conditions. Lower the *P*-value, more significant were the GO term and KEGG pathway.

The results revealed that 1904 LncRNAs were differentially expressed between health controls and active TB patients (Figure 3). As compared to the control group, the active pulmonary TB group showed up-regulation in 1081 lncRNAs and down-regulation in 823 lncRNAs, among which, the top 10 lncRNAs that were up- and down-regulated are shown in Table 2. NONHSAG003987 was the most upregulated lncRNA gene among them, while linc-SNAP25-1 was the most downregulated gene. The top 10 mRNAs that were up- and down-regulated are listed in Table 3. The most upregulated mRNA gene among them was CHI3L1, whereas the most downregulated gene was FAM177B.

**TABLE 3:** The top 10 upregulated and downregulated mRNAs in the PBMCs of ATB patients, compared to the HC group.

mRNA gene symbol	Regulation	Fold change	*P*-value
CHI3L1	Up	118.88714	0.000487
IL1R2	Up	102.415306	0.000145
KCNJ15	Up	94.443924	0.000000753
CYP4F2	Up	90.364334	0.000926
MME	Up	90.1744	0.00023
MGAM	Up	85.16178	0.000177
TNFRSF10C	Up	76.06052	0.00128015
IFIT3	Up	73.37543	0.002505481
CXCL1	Up	69.12307	0.004915247
CYP4F3	Up	67.52758	0.0000468
FAM177B	Down	294.74496	0.004868665
MADCAM1	Down	17.573362	0.005767919
CSAG2	Down	15.699993	0.013869465
FCRL2	Down	11.852164	0.012213992
CABLES1	Down	11.157983	0.013856612
C10orf114	Down	10.827955	0.00771278
PRR20B	Down	10.800922	0.002674374
NOS2	Down	10.639506	0.002279884
FCRL1	Down	10.294664	0.003636744
GSTM5	Down	10.257128	0.01034451

### Functional prediction results of the differentially expressed lncRNAs

Gene Ontology (GO)^1^ and Kyoto Encyclopedia of Genes and Genomes (KEGG)^2^ pathway analyses of the dysregulated mRNAs were performed to elucidate the functions of lncRNAs, with separate analyses conducted for upregulated and downregulated mRNAs. Initially, we identified the co-expressed mRNAs for each differentiated lncRNA and conducted a functional enrichment analysis on this group. The predicted functional term for a specific lncRNA was derived from the enriched functional terms. Co-expressed lncRNA mRNAs were discovered using Pearson Correlation calculations with correlation *P*-values < 0.05, using R Studio. Subsequently, we employed the hypergeometric cumulative distribution function to determine the enrichment of functional terms in the annotation of co-expressed mRNAs.

The results indicated that PBMCs from TB patients significantly differed from healthy controls in terms of biological processes and signaling pathways. Based on the GO analysis, the majority of biological processes featuring dysregulated lncRNAs were primarily associated with neutrophil activation, immune responses involving neutrophil activation, neutrophil-mediated immunity, and neutrophil degranulation. In contrast, the KEGG analysis revealed that downregulated mRNAs were associated with neuroactive ligand-receptor interaction and olfactory transduction, while upregulated lncRNAs were primarily enriched in neutrophil extracellular trap formation, the chemokine signaling pathway, and FcgammaR-mediated phagocytosis in TB patients. Figure 3 depicts the key functional clusters.

The results indicate that the response of PBMCs during active TB infection is characterized by variable regulation and suppression.

### Correlation analysis of lncRNAs and mRNAs

We constructed a lncRNA-mRNA interaction network to explore the regulatory mechanisms of lncRNAs in PTB. We first calculated the Pearson Correlation between the expression values of each lncRNA and each mRNA. Then, we combined the data of differentially expressed (DE) mRNAs and DE lncRNAs after performing correlation analysis on the lncRNA-mRNA pair.

**FIGURE 4:**
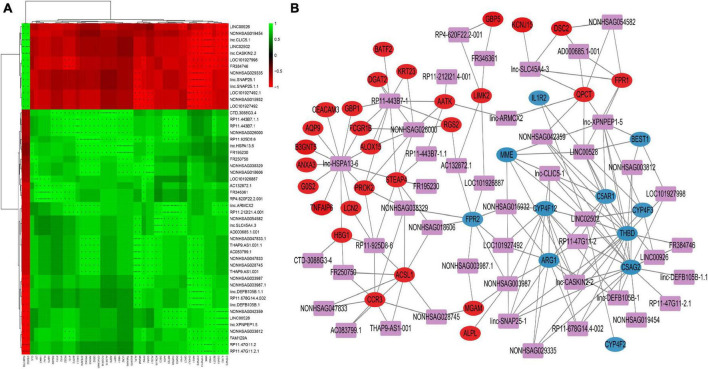
Correlation analysis of lncRNA and mRNA **(A)** Correlation heatmap. The horizontal axis represents lncRNA, the vertical axis represents mRNA, the green square represents positive correlation, and the red square represents negative correlation, “+” stands for FDR < 0.1, “ + ⁣ + “ stands for FDR < 0.01, “ + ⁣ + ⁣ + “ stands for FDR < 0.001. **(B)** DE lncRNAs and DE mRNAs co-expression network. The networks were based on lncRNA–mRNA interactions. In the network, purple squares represent lncRNAs, which are located in the center of the network, and circles represent significantly associated mRNAs, which are distributed around lncRNAs. The red circles represent significantly positively associated mRNAs and the blue circles represent significantly negatively associated mRNAs.

For correlation analysis, we used a total of 198 DE lncRNAs and 392 DE mRNAs obtained from our data. The lncRNA-mRNA interaction network was built according to the interaction mechanism of the two molecules. As a result, we discovered a total of 469 lncRNA-mRNA interactions, including 239 mRNAs and 64 lncRNAs (Figure 4).

This network provides valuable insights into the regulatory roles of lncRNAs in PTB. The identified interactions can help researchers understand the molecular pathogenesis of PTB and potentially identify new therapeutic targets for the treatment of the disease. Additionally, these networks can be used to further study the functional roles of lncRNAs in other biological processes and pathological conditions.

### qRT-PCR validation

To validate the microarray results, we selected eight lncRNAs for real-time quantitative polymerase chain reaction (QRT-PCR) examination across all samples. The selected lncRNAs were LINC00528, lnc-SLC45A4-3, LINC00926, lnc-XPNPEP1-5, lnc-CASKIN2-2, lnc-HSPA13-6, lnc-CLIC5-1, and LINC02502.

The results showed that the expression of lncRNAs LINC00528, lnc-SLC45A4-3, and LINC00926 was significantly higher in both the latent infection group and the TB patient group compared to the uninfected HC group (*P*-value < 0.05). On the other hand, the expression of lncRNAs lnc-XPNPEP1-5, lnc-CASKIN2-2, lnc-HSPA13-6, lnc-CLIC5-1, and LINC02502 was significantly reduced in both the latent infection group and the TB patient group compared to the uninfected HC group (all *P*-values < 0.05).

**FIGURE 5:**
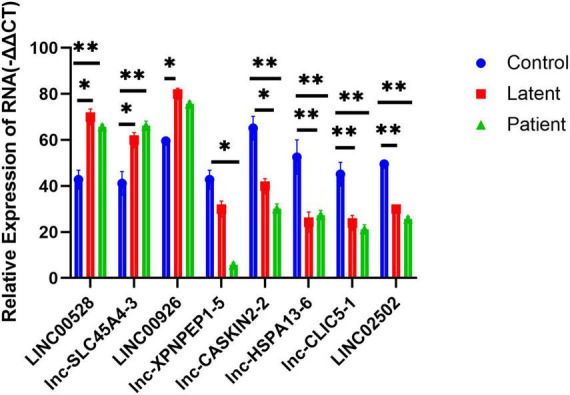
Quantitative PCR (QRT-PCR) validation of eight differentially expressed lncRNAs and lncRNA as well as its nearby protein-coding gene. Eight lncRNAs LINC00528, lnc-SLC45A4-3, LINC00926, lnc-XPNPEP1-5, lnc-CASKIN2-2, lnc-HSPA13-6, lnc-CLIC5-1, and LINC02502 were chosen for the validation of the gene chip results using qRT-PCR. LINC00528, lnc-SLC45A4-3, and LINC00926 were upregulated, while lnc-XPNPEP1-5, lnc-CASKIN2-2, lnc-HSPA13-6, lnc-CLIC5-1, and LINC02502 were downregulated in the latent infection group (*n* = 50) and TB patient group (*n* = 50) relative to health controls (*n* = 50). *Response that is significantly different from the HC group (*p* < 0.05). **Response that is significantly different from the HC group (*p* < 0.01).

The findings from the QRT-PCR analysis were consistent with the microarray data, supporting the reliability of our results. This validation process strengthens the evidence for the regulatory roles of these lncRNAs in PTB, providing a basis for further research into their functional roles and potential therapeutic targets (Figure 5).

## Discussion

In the present study, we employed microarray technology to examine the mRNA and lncRNA expression profiles of peripheral blood mononuclear cells (PBMCs) from tuberculosis (TB) patients and healthy individuals. This was followed by bioinformatics analysis to delve into the functional networks and potential regulatory roles of the differentially expressed lncRNAs and mRNAs. To corroborate our findings, we also used quantitative real-time polymerase chain reaction (qRT-PCR). Through microarray analysis, we were able to compare the lncRNA expression profiles of TB patients and healthy controls. The bioinformatics analysis revealed several lncRNAs and mRNAs with altered expression in PBMCs from TB patients, suggesting that these aberrant long non-coding RNAs (lncRNAs) may play crucial roles in the pathophysiology and progression of tuberculosis. We further identified several dysregulated biological processes in human PBMCs, including “neutrophil extracellular trap formation,” “Chemokine signaling pathway,” “Fc gamma R-mediated phagocytosis,” “Neuroactive ligand-receptor interaction,” and “AMPK signaling pathway,” using KEGG pathway-based paired-sample analysis. The majority of these dysregulated pathways are essential for the onset and progression of tuberculosis.

Neutrophils play a significant role in the pathogenesis of TB, as they are rapidly recruited to the infection site and capture *Mycobacterium tuberculosis (Mtb)* in extracellular traps (NETs) (Borkute et al., 2021; Pellegrini et al., 2021). This occurs due to the release of neutrophil extracellular traps (NETs), which contain antigens that can prime T cells and exacerbate inflammation. Moreover, neutrophils can modulate the immune response by interacting with other immune cells, such as macrophages, dendritic cells, and T cells. Neutrophils have been shown to generate NETs in response to various microbes, TLRs, Fc receptors, chemokine and cytokine receptors, as well as in response to stimulating substances like phorbol myristate acetate (PMA) (Braian et al., 2013; Ravindran et al., 2019). Chemokines produced in response to *Mtb* infection have been shown to efficiently facilitate the trafficking of dendritic cells (DCs) to the lymph nodes, the attraction of activated T cells to the lung, and the proper positioning of T cells within the lung parenchyma to mediate optimal *Mtb* control (Monin and Khader, 2014). These chemokine-dependent pathways facilitate the inhibition of *Mtb* development, although they often do not eradicate the bacteria entirely (Park et al., 2021). The design of vaccines and adjuvants can be significantly improved by gaining a deeper understanding of the mechanisms underlying *Mtb* containment, which will also contribute to the development of novel anti-TB medicines. Several studies have identified Mtb lncRNAs that may be involved in regulating the immune response, including those that modulate the function of neutrophils. Studis reported that Mtb-lncRNA, a lncRNA encoded by the Mtb genome, regulates the expression of various Mtb genes involved in metabolism, virulence, and stress response. This lncRNA has also been shown to modulate the expression of pro-inflammatory cytokines in macrophages and neutrophils, suggesting that it may play a role in regulating the immune response against Mtb.

In the present study, we rigorously controlled the quality of the experimental data. This entailed examining the integrity, purity, and concentration of RNA to ensure the accuracy and repeatability of the experimental procedures. We employed multiple bioinformatic methods to corroborate our findings and elucidate the functional and regulatory relationships of the obtained results in the context of biology. By comparing our results with those of other published studies, we determined whether there was consistency among them. We also validated the differentially expressed lncRNAs through various experiments, such as real-time fluorescent quantitative PCR, to confirm our findings (Song et al., 2019; Huang et al., 2020).

Based on the identified dysregulated pathways, we discovered eight PBMC lncRNAs that displayed different expression patterns in TB patients, thereby creating an 8-lncRNA TB biological signature. This signature was also found to be capable of distinguishing TB patients from healthy controls in an independent cohort. The significant changes in pathway or function which are related Neutrophils suggest that the findings show a strong neutrophil response, which could be due to an infection or inflammation in the sample. The further investigate of the specific pathways and functions is needed to gain a deeper understanding of the neutrophil response and potential causes. Our research clearly suggests that PBMC lncRNAs hold potential as biomarkers for differentiating active TB patients from healthy individuals. For instance, lncRNA LINC00528 functions as a predictive biomarker for myocardial infarction (MI) (Sheng et al., 2020). It regulates myocardial infarction (MI) through the miR-143-3p/COX-2 axis (Liu et al., 2020). LINC00528 is significantly associated with immunological activity, and its target mRNAs are involved in biological processes triggered by various immune cells, including T cells, lymphocytes, and leukocytes. The pathways that positively and negatively regulate leukocyte cell-cell adhesion are highly enriched in LINC00528, which includes multiple molecules of the CD family (Zhang et al., 2021). This indicates that when *Mtb* invades the host, LINC00528 is closely linked to the immunological control of lymphocytes. Another recent study revealed that LINC02502 is directly related to calcium accumulation in mitochondria, suggesting that LINC02502 is connected to TB patients’ energy metabolism (Guo et al., 2022).

This study demonstrates a correlation between LINC02502 and the energy metabolism of TB patients. The lncRNA LINC00926 negatively regulates the expression of phosphoglycerate kinase 1 (PGK1) and predicts a favorable clinical outcome for breast cancer. This regulation occurs through an increase in PGK1 ubiquitination, which is mediated by the E3 ligase STUB1 (Chu et al., 2021; Guo et al., 2022). The WNT signaling pathway, crucial for various physiological processes such as cell fate determination, development, differentiation, and more, is dysregulated in PTSD and contributes to the upregulation of pro-inflammatory genes. The promoter of WNT10B’s H3K4me3 is regulated by LINC00926, leading to enhanced WNT signaling (Bam et al., 2022). Our study’s Gene Set Enrichment Analysis (GSEA) revealed an enrichment of the WNT signaling pathway. Although it is unknown whether elevated WNT10B expression in PBMCs leads to additional lung parenchymal inflammation in TB patients, this research suggests that LINC00926 could serve as a potential TB biomarker and therapeutic target. Unfortunately, most DE lncRNAs, including lnc-SLC45A4-3, LINC00926, lnc-XPNPEP1-5, lnc-CASKIN2-2, lnc-HSPA13-6, and lnc-CLIC5-1, have not yet been investigated; further research is warranted.

In summary, we identified and validated distinct DElncRNAs in TB cases. Our findings suggest that the genes LINC00528, LINC00926, and LINC02502 may play a significant regulatory role in the development of TB. These discoveries provide insights into the etiology of immunoregulatory dysfunction in tuberculosis and could reveal potential molecular pathways and therapeutic targets.

## Data availability statement

The datasets presented in this study can be found in online repositories. The names of the repository/repositories and accession number(s) can be found below: GSA–PRJCA019204.

## Ethics statement

The studies involving human participants were reviewed and approved by Ethics Review Committee of Jiangsu Provincial Center for Disease Control and Prevention [Ethics Authorization Number: JSCDCLL(2014)003]. The studies were conducted in accordance with the local legislation and institutional requirements. The participants provided their written informed consent to participate in this study.

## Author contributions

YL: Conceptualization, Data curation, Formal analysis, Funding acquisition, Investigation, Methodology, Project administration, Resources, Software, Supervision, Validation, Visualization, Writing – original draft, Writing – review and editing. ZF: Data curation, Investigation, Writing – original draft. GL: Methodology, Formal analysis, Writing – original draft, Writing – review and editing. HS: Formal analysis, Investigation, Methodology, Writing – original draft. YW: Methodology, Supervision, Writing – review and editing. LZ: Funding acquisition, Investigation, Methodology, Resources, Supervision, Visualization, Writing – review and editing.

## Footnotes

1
https://geneontology.org/

2
https://www.genome.jp/kegg/
